# Supplementary material for: Antimicrobial use in laboratory rodent facilities in Australia and New Zealand- a cross-sectional survey of veterinarians and facility managers
Source: PLoS One. 2024 Aug 23;19(8):e0292908. doi: 10.1371/journal.pone.0292908 (PMC11343402; doi:10.1371/journal.pone.0292908)
Supplement: Attachment — Submitted filename: Response to Reviewers.docx [file pone.0292908.s003.docx]

18/04/2024

To whom it may concern,

We are delighted with significance of Reviewer #1’s comment: *“Most of the animal facilities dispose the biomedical waste as per the local rules. Hence, residual effect of the antibiotics in environment needs to be elaborated considering the half-life of the drug, concentration etc., in the Discussion section.”* Environmental contamination is one of the most concerning findings of this study.

Within the Discussion section, we have elaborated on this matter, including the specific environmental half-lives of the most used antimicrobials in laboratory rodents, according to this survey’s respondents. The concentrations in the environment are unknown, from this source, as there are no data or studies that can be found, and we have acknowledged this knowledge gap. We have also expanded on the issue as a newly acknowledged driver of antimicrobial resistance, and environmental pollution, along with the pathways by which human and animal health may be affected by these disposal practices.

We have completed and attached the Inclusivity in Global Research Questionnaire and amended both the tracked and clean manuscripts to reflect requested changes, and uploaded these.

All changes have been referenced with peer reviewed literature.

Thank you for your consideration,

Dr Wilcox
